# Mechanism of hydroxysafflor yellow A on acute liver injury based on transcriptomics

**DOI:** 10.3389/fphar.2022.966759

**Published:** 2022-09-02

**Authors:** Xiangmei Hou, Ziying Zhang, Yuehong Ma, Rong Jin, Bing Yi, Dongdong Yang, Lijie Ma

**Affiliations:** Inner Mongolia Key Laboratory of Molecular Biology, Inner Mongolia Medical University, Hohhot, China

**Keywords:** acute liver injury, hydroxysafflor yellow A, RNA-sequencing, differentially expressed genes, targets

## Abstract

**Objective:** To investigate how Hydroxysafflor yellow A (HSYA) effects acute liver injury (ALI) and what transcriptional regulatory mechanisms it may employ.

**Methods:** Rats were randomly divided into five groups (*n* = 10): Control, Model, HSYA-L, HSYA-M, and HSYA-H. In the control and model groups, rats were intraperitoneally injected with equivalent normal saline, while in the HSYA groups, they were also injected with different amounts of HSYA (10, 20, and 40 mg/kg/day) once daily for eight consecutive days. One hour following the last injection, the control group was injected into the abdominal cavity with 0.1 ml/100 g of peanut oil, and the other four groups got the same amount of a peanut oil solution containing 50% CCl_4_. Liver indexes were detected in rats after dissection, and hematoxylin and eosin (HE) dyeing was utilized to determine HSYA’s impact on the liver of model rats. In addition, with RNA-Sequencing (RNA-Seq) technology and quantitative real-time PCR (qRT-PCR), differentially expressed genes (DEGs) were discovered and validated. Furthermore, we detected the contents of anti-superoxide anion (anti-O_2_^−^) and hydrogen peroxide (H_2_O_2_), and verified three inflammatory genes (Icam1, Bcl2a1, and Ptgs2) in the NF-kB pathway by qRT-PCR.

**Results:** Relative to the control and HSYA groups, in the model group, we found 1111 DEGs that were up-/down-regulated, six of these genes were verified by qRT-PCR, including Tymp, Fabp7, Serpina3c, Gpnmb, Il1r1, and Creld2, indicated that these genes were obviously involved in the regulation of HSYA in ALI model. Membrane rafts, membrane microdomains, inflammatory response, regulation of cytokine production, monooxygenase activity, and iron ion binding were significantly enriched in GO analysis. KEGG analysis revealed that DEGs were primarily enriched for PPAR, retinol metabolism, NF-kB signaling pathways, etc. Last but not least, compared with the control group, the anti-O_2_^−^ content was substantially decreased, the H_2_O_2_ content and inflammatory genes (Icam1, Bcl2a1, and Ptgs2) levels were considerably elevated in the model group. Compared with the model group, the anti-O_2_^−^ content was substantially increased, the H_2_O_2_ content and inflammatory genes (Icam1, Bcl2a1, and Ptgs2) levels were substantially decreased in the HSYA group (*p* < 0.05).

**Conclusion:** HSYA could improve liver function, inhibit oxidative stress and inflammation, and improve the degree of liver tissue damage. The RNA-Seq results further verified that HSYA has the typical characteristics of numerous targets and multiple pathway. Protecting the liver from damage by regulating the expression of Tymp, Fabp7, Serpina3c, Gpnmb, Il1r1, Creld2, and the PPAR, retinol metabolism, NF-kappa B signaling pathways.

## 1 Introduction

Acute liver injury (ALI) is associated with a rapid reduction in liver enzymes (Tao et al., 2021), and it’s mechanism involves a complicated interaction of hepatocyte degeneration, inflammatory reaction, reactive oxygen species (ROS), necrosis and apoptosis of hepatocytes (Yang et al., 2021). ALI is usually caused by viral infections (Marra et al., 2021), drugs or alcohol abuse (Oh and Park, 2015), and ingestion of toxic substances (Allard et al., 2019). Liver disease is a major health problem with a very poor prognosis, severe hepatic damage might result in liver failure or death (Åberg et al., 2018). Currently, there are no effective targeted drugs. Consequently, it is crucial to identify critical molecular targets for ALI prevention. Hydroxysafflor yellow A (HSYA), one of the most important active elements of safflower (Guo et al., 2019), is mainly used in clinical for coronary heart disease, cerebral ischemia-reperfusion, hyperlipidemia and other diseases (Han et al., 2016; Zou et al., 2018; Tan et al., 2020). It is reported that HSYA has the ability to neutralize oxygen free radicals, prevent lipid peroxidation and reduce inflammatory response (Li et al., 2017). Other studies have shown that HSYA has numerous biological roles, including anti-tumor, regulating glucose, lipid metabolism, anticoagulation, and antihypertensive (Ao et al., 2018). However, the mechanism related to the effect of HSYA on ALI is not clear. Our previous research showed that safflower and compound preparations with it had a very good mitigation effect on ALI (LV et al., 2018; Lu et al., 2021), we suspect that HSYA is one of the main protective components in safflower.

In recent years, RNA-Sequencing (RNA-Seq) has developed rapidly, and with it’s advantages of high reproducibility, accuracy, and extensiveness (Jiang et al., 2015), it has gradually become a powerful tool for detecting genome-wide transcriptional patterns such as differentially expressed genes (DEGs), signaling pathways, novel transcripts, and disease biomarkers in complex tissues or cells (Tran et al., 2015; Zang et al., 2019). Rats were used to generate an ALI model caused by carbon tetrachloride (CCl_4_) in this investigation, and the Illumina NovaSeq 6000 high-throughput sequencing technology was used to construct the corresponding transcriptome library. Then the differential expression analysis of genes and the functional annotation of GO and KEGG were carried out. We attempted to estimate the associated targets of HSYA on the protective impact of CCl_4_-induced ALI rats and explore the associated mechanisms, providing a theoretical foundation for future investigation.

## 2 Materials and methods

### 2.1 Main instruments and reagents

Automatic chemistry analysis instrument (Beckman AU5800, BECKMAN COULTER, United States). HSYA (purity > 98%) was obtained from Chengdu Zhibiao Pure (Chengdu, China). The ALT, AST, ALP, SOD, MDA, Hydrogen peroxide, Inhibition and produce superoxide anion assay kits were from Nanjing Jiancheng (Nanjing, China). TNF-α, IL-6, and IL-1β assay kits were from Wuhan Gene Beauty (Wuhan, China). TRIzol reagent, FastKing RT Kit, SYBR Premix Ex Taq were purchased from Tiangen (Beijing, China). Other reagents possessed a higher degree of analytical purity.

### 2.2 Animals and treatment

SPF-grade Wistar rats (male, 180–220 g) were obtained from SPF Biotechnology Co., Ltd. (Beijing, China). The license number for Animal Production: SCXK (Beijing) 2019-0010. All animals were raised in the Laboratory Animal Center of Inner Mongolia Medical University (20°C ± 2°C, 12 h day/night cycle). Before beginning studies, animals were given adequate water and food for 5 days to adapt to the animal center’s environment. The experiments were conducted according to the Animal Welfare Guidelines and approved by the Inner Mongolia Medical University’s Animal Care and Use Committee (No. YKD202001020). These animals were arbitrarily separated into five categories (*n* = 10): Control, Model, HSYA-L, HSYA-M, and HSYA-H. The modeling method was as follows: in the control and model groups, rats were intraperitoneally injected with equivalent normal saline, and rats in HSYA groups were also injected with different amounts of HSYA (10, 20, and 40 mg/kg/day) once daily for eight consecutive days. One hour following the last injection, the control group was injected into the abdominal cavity with 0.1 ml/100 g of peanut oil. Meanwhile, the other four groups got the same amount of a peanut oil solution containing 50% CCl_4_. After 24 h, 150 mg/kg of sodium pentobarbital *via* intraperitoneally to euthanize the rats, and serum and liver tissues were collected for subsequent analysis.

### 2.3 Calculation of body mass, wet liver weight, and liver index

After collecting blood samples, rats were killed. The whole liver was immediately removed and put in ice-cold PBS, and then we wiped off the water on the liver surface with filter paper to calculate the liver index. Liver Index = Wet Liver Weight (g)/Body Mass (g) × 100%.

### 2.4 Biochemical analysis

Using an automated biochemical analyzer and biochemical assay kits, ALT, AST, and ALP levels were determined.

### 2.5 Enzyme-linked immunosorbent assay

The right lobe tissues of rats in all groups were washed with normal cold saline, and 10% liver homogenate was prepared. The supernatant was taken by centrifugation (4°C, 12,000 r/min, and 15 min), and the SOD activity, MDA and inflammatory mediators (TNF-α, IL-1β, and IL-6) levels were measured as per directions for kits.

### 2.6 Liver histopathological examination

The right liver tissues of rats for each group were preserved in 10% buffered formalin. The tissues were dehydrated, wax-impregnated, fixed, sliced (4–5 m thick), with hematoxylin-eosin staining (HE), and viewed under a light microscope for histological liver alterations. The ratio between the necrotic area of tissue and the whole visual field was calculated using Image-Pro Plus 6.0 software.

### 2.7 Transcriptome sequencing

#### 2.7.1 Total RNA extraction

Three liver samples were randomly selected from the control, model, and HSYA-M groups (Note: the HSYA group mentioned in the sequencing section only represented the HSYA-M). In each group, the liver’s left region was removed. After extracting total RNA, Agilent 2100 bioanalyzer was used to accurately detect the concentration, total amount, and integrity of RNA.

#### 2.7.2 Library preparation, quality inspection, and sequencing analyses

Oligo (dT) magnetic beads were used to enrich PolyA-tailed mRNA, which was subsequently fragmented. The first strand of cDNA was produced using fragmented mRNA and random oligonucleotide, whereas the second strand was generated by DNA polymerase I, RNase H, and dNTP. After adding purified cDNA to poly (A), Utilizing AMPure XP beads, a fragment of 300 bp was chosen for PCR amplification. PCR products were refined, and the final library was obtained. Moreover, using the Qubit 2.0 Fluorometer and Agilent 2100 Bioanalyzer, the filtered sequences were assessed. Finally, on the Illumina NovaSeq 6000 equipment, 150-bp partnered reads were used for the sequencing. These processes were operated by Beijing novogene Bioinformatics Technology Co., Ltd.

#### 2.7.3 Gene quality control

To obtain the clean reads with high quality, the sequencing quality was assessed by fastp (version 0.19.7) software to remove the adaptor sequence, reads from N (N indicates that the data cannot be verified), which was >10% and poor quality reads which was >50% bases from raw reads. Using the HISAT2 program, the positional information of Reads in the reference genome was acquired.

#### 2.7.4 Differential expression analysis

The quantitative study of gene expression was performed using the FeatureCount tool, and FPKM > 1 was used to define gene expression. The DEGs comparison across two groups was made using DESeq2 software, and DEGs with differential expression multiples (|log_2_FC| ≥ 1) and *p* < 0.05 were considered significantly DEGs. ClusterProfiler software was used to examine the enrichment of GO and KEGG. *p* < 0.05 was deemed statistical significance.

#### 2.7.5 Real-time quantitative PCR analysis

For establishing the validity of RNA-Seq, DEGs were analyzed by qRT-PCR, GAPDH was used to standardize each sample. The kit’s instructions were followed for total RNA extraction, inverse transcription, and qRT-PCR tests. Conditions for amplification were 95°C for 15 min, cycling at 95°C for 10 s, 60°C for 32 s, repeated 40 times. The 2^−△△CT^ method was used for quantification and analysis. Experiments were repeated three times. Sangon Biotech (Shanghai, China) synthesized the Primer combinations seen in Table 1.

**TABLE 1:** Primer sequences of DEGs to be measured.

Gene	Forward primer	Reverse primer
Tymp	CCC​TGG​AAG​TGG​AAG​AAG​CGT​TG	CTG​GTC​TTG​GGT​TTC​TGC​CTG​TC
Fabp7	TGT​GAT​TCG​GTT​GGA​TGG​AGA​CAA​G	CAT​AAC​AGC​GAA​CAG​CAA​CGA​CAT​C
Serpina3c	GCT​GTC​CTC​TGT​GAT​GGC​ATA​CTG	TGT​GAA​GTC​AGT​GTT​GAT​GGA​AGC​C
Gpnmb	AGA​TGC​CAG​AAG​GAA​GAT​GCC​AAC	GGG​TCA​GAA​GCC​AGC​TCC​AAA​TC
Il1r1	CTG​CCG​AGG​CTT​GTG​ACA​TCT​TC	CGA​CAG​CAG​AGG​CAC​AAT​GAG​AC
Creld2	AGA​GGA​ACG​AGA​CCC​ACA​GCA​TC	GCC​GCA​CAC​TCA​TCT​ACA​TCC​AC
Icam1	TGT​CGG​TGC​TCA​GGT​ATC​CAT​CC	TTC​GCA​AGA​GGA​AGA​GCA​GTT​CAC
Bcl2a1	ATC​CAC​TCC​CTG​GCT​GAG​AAC​TAT​C	AAA​GCA​ACT​CTC​TGT​AGC​ACT​CTG​G
Ptgs2	CAC​ATT​TGA​TTG​ACA​GCC​CAC​CAA​C	AGT​CAT​CAG​CCA​CAG​GAG​GAA​GG
GAPDH	TCA​CCC​CAT​TTG​ATG​TTA​GCG	GCA​AGT​TCA​ACG​GCA​CAG​TCA

### 2.8 Detection of anti-superoxide anion and hydrogen peroxide

To further determine the effects of HSYA against oxidative stress induced by CCl_4_, we have determined the contents of anti-superoxide anion (anti-O_2_^−^) and hydrogen peroxide (H_2_O_2_) in liver tissue of rats in each group, the specific operation was carried out according to the kits’ instructions.

### 2.9 Statistical analysis

Using the GraphPad prism8 tool, statistical analyses were conducted, the tests were provided as means ± standard deviation ( $\bar{x}$ ± *s*), the number of samples is represented by *n*. If the data satisfied the homogeneity tests of normality and variance, using One-way ANOVA, several groups were evaluated, while pairwise comparisons were conducted by the LSD test. If the data only conformed or did not conform to the normal distribution and/or did not satisfy the homogeneity test of variance, a rank-sum test was used to test rank count data. *p* < 0.001, *p* < 0.01, and *p* < 0.05 showed statistical validity.

## 3 Results

### 3.1 Effects of hydroxysafflor yellow A on liver morphology of CCl_4_-induced acute liver injury in rats

**FIGURE 1:**
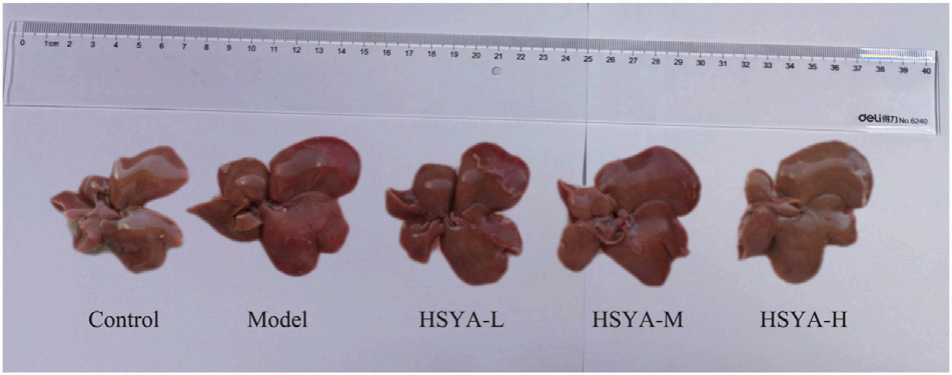
Morphology of liver tissues in each group.

As seen in Figure 1, in the control group, the hepatic of rats was bright red, and the membrane surface was smooth and soft. In contrast to the control group, in the model group, significantly greater liver volume was witnessed, the surface of capsule was rough, the texture was hard, and local rotten. Relative to the model group, the hepatic volume in HSYA groups decreased slightly smaller, the liver edge was slightly sharper, the surface was slightly rougher, the texture was slightly harder. The overall state was among the control and model groups.

### 3.2 Effects of hydroxysafflor yellow A on body mass, wet liver weight, and liver index

**FIGURE 2:**
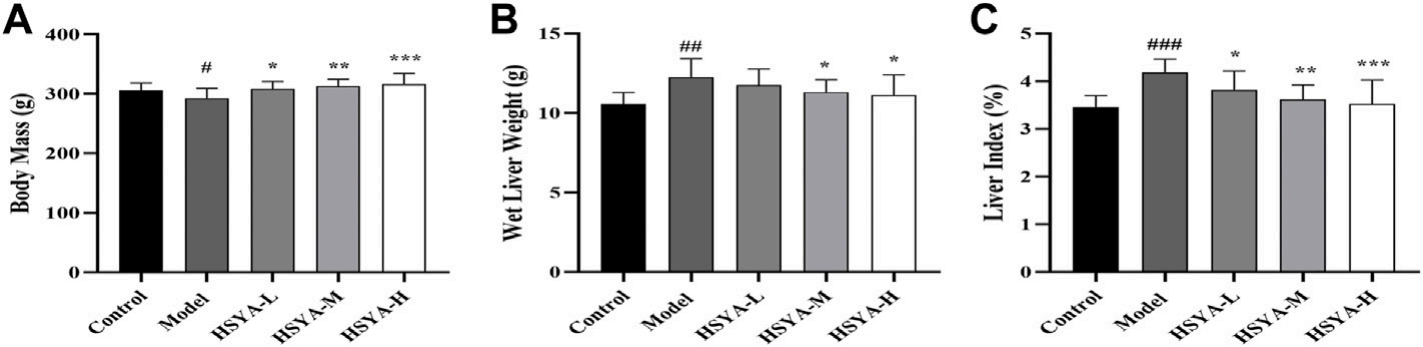
Effects of HSYA on Body Mass, Wet Liver Weight and Liver Index. **(A)** Body Mass; **(B)** Wet Liver weight; **(C)** Liver Index ( $\bar{x}$ ± *s*, *n* = 10). ^#^ *p* < 0.05, ^###^ *p* < 0.001 vs. the control group; *p < 0.05, **p < 0.01 vs. the model group.

Weight loss and liver index increase might indicate liver damage (Li et al., 2019). As observed in Figure 2, relative to the control group, in the model group, the body mass of rats was dramatically decreased (*p* < 0.05). Relative to the model group, there was no significant change in the HSYA-L, but the body mass of rats was dramatically increased in the HSYA-M and HSYA-H groups (*p* < 0.01, *p* < 0.05). CCl_4_ significantly increased rats’ wet liver weight and liver index (*p* < 0.001). Relative to the model group, the wet liver weight and liver index of rats were greatly diminished in the HSYA-M and HSYA-H groups (*p* < 0.01, *p* < 0.05). The results showed that HSYA could effectively protect the liver.

### 3.3 Effects of hydroxysafflor yellow A on biochemical indicators

**FIGURE 3:**
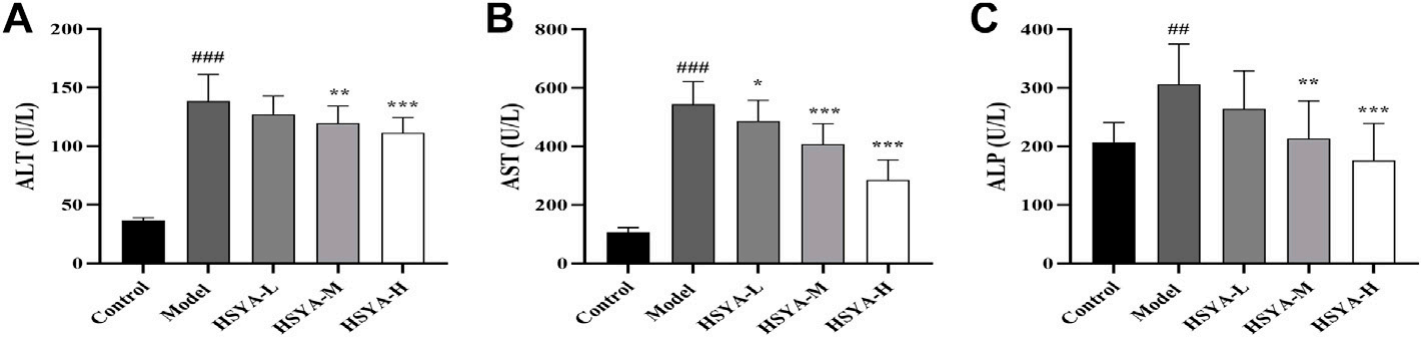
Effects of HSYA on Biochemical Indicators. **(A)** ALT; **(B)** AST; **(C)** ALP ( $\bar{x}$ ± *s*, *n* = 10). ^###^ *p* < 0.001 vs. the control group; *p < 0.05, **p < 0.01, ***p < 0.001 vs. the model group.

ALT, AST, and ALP are valuable biochemical indicators for examining early hepatotoxicity (Ali et al., 2021). As seen in Figure 3, relative to the control group, in the model group, the ALT, AST, and ALP activities were dramatically increased (*p* < 0.001, *p* < 0.01). In comparison with the model group, in the HSYA-M and HSYA-H groups, the ALT and ALP activities were considerably reduced (*p* < 0.001, *p* < 0.01), and the AST activity in all HSYA groups were also obviously decreased (*p* < 0.001, *p* < 0.05). The above revealed that HSYA could prevent ALI caused by CCl_4_.

### 3.4 Effects of hydroxysafflor yellow A on oxidative indexes and expression levels of inflammatory factors

**FIGURE 4:**
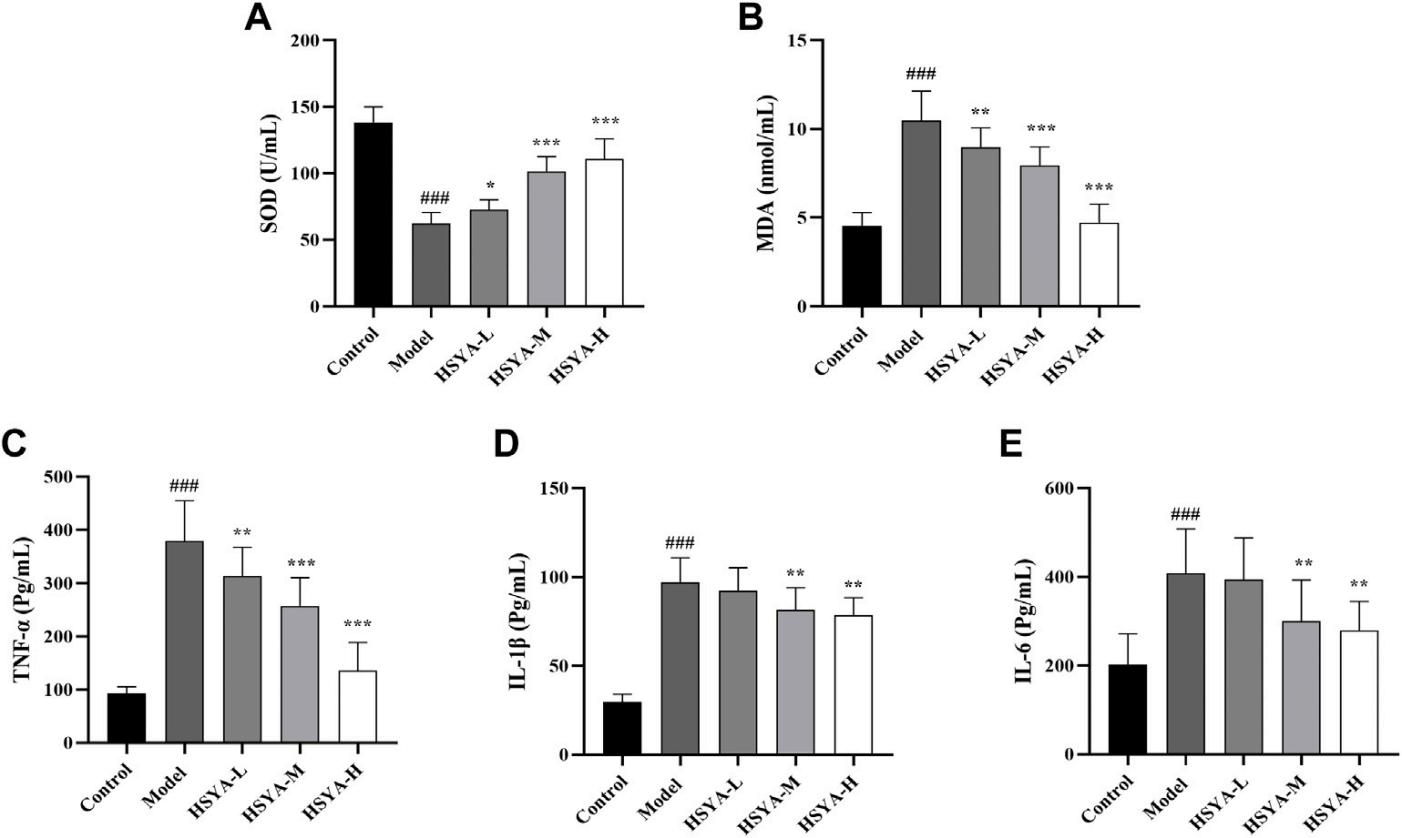
Effects of HSYA on oxidative indexes and expression levels of inflammatory factors. **(A)** SOD; **(B)** MDA; **(C)** TNF-α; **(D)** IL-1β; **(E)** IL-6 ( $\bar{x}$ ± *s*, *n* = 10). ^###^ *p* < 0.001 vs. the control group; *p < 0.05, **p < 0.01, ***p < 0.001 vs. the model group.

Oxidative stress and inflammation are strongly associated with the pathophysiology of ALI (Al-Dossari et al., 2020). To analyze HSYA’s effects on CCl_4_-induced hepatic lipid peroxidation and inflammatory response, we detected the SOD activity, MDA and inflammatory factors (TNF-α, IL-1β, and IL-6) expression levels. As seen in Figure 4, relative to the control group, the SOD activity in liver tissues was dramatically reduced, and the content of MDA and inflammatory factors levels were considerably elevated in the model group (*p* < 0.001). Relative to the model group, the SOD activity in liver tissues of all HSYA groups was dramatically increased, MDA and TNF-α levels in all HSYA groups were drastically reduced (*p* < 0.001, *p* < 0.01, and *p* < 0.05), IL-1β and IL-6 levels in HSYA-M and HSYA-H groups were also noticeably diminished (*p* < 0.01). These findings demonstrated that HSYA might decrease oxidative stress and inflammatory responses generated by CCl_4_.

### 3.5 Effects of hydroxysafflor yellow A on the liver tissues pathology

**FIGURE 5:**
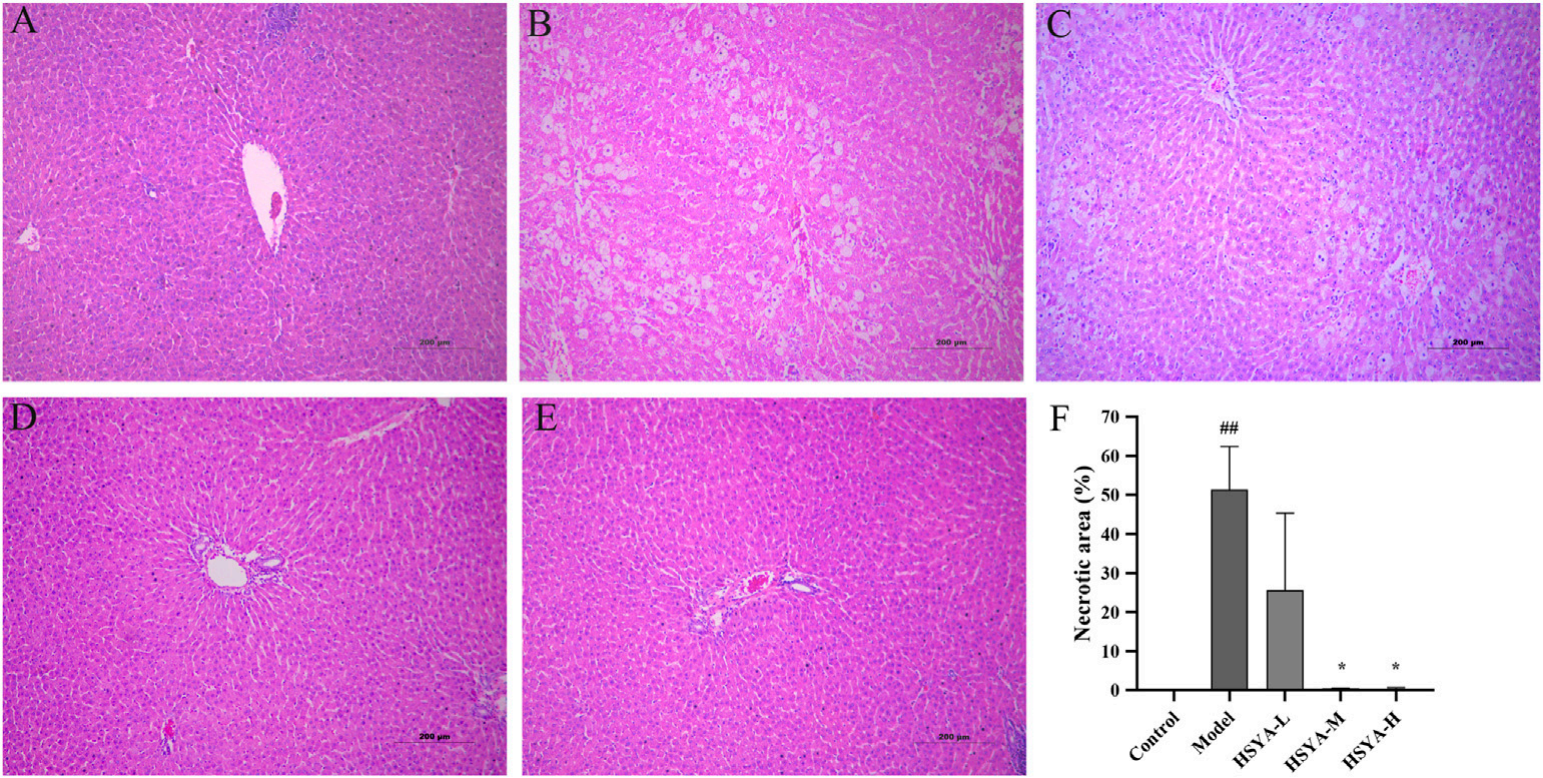
Effects of HSYA on the liver tissue pathology. **(A)** Control; **(B)** Model; **(C)** HSYA-L; **(D)** HSYA-M; **(E)** HSYA-H; **(F)** Hepatocyte necrosis quantified ( $\bar{x}$ ± *s*, *n* = 3). ^##^ *p* < 0.01 vs. the control group; *p < 0.05 vs. the model group.

As seen in Figure 5, the hepatic cords of rats in the control group were neatly arranged, the hepatic lobules and nucleus were visible without obvious inflammation, cell degeneration and necrosis (Figure 5A). In the model group, the hepatic cords were obviously disordered, the hepatic lobule structure mostly was destroyed, the surrounding liver tissues showed vacuolar degeneration, the major vein was surrounded by infiltrating inflammatory mediators, and apoptotic bodies were standard (Figure 5B). In the HSYA-L group, the hepatic cords of rats were disordered, the boundary of hepatic lobules was not clear, the liver cells showed obvious degeneration and necrosis, moderate diffuse vacuolar degeneration, and inflammatory infiltration was lighter than that in the model group (Figure 5C). In the HSYA-H group, the hepatic cords of rats were slightly disordered, it was easy to see the structure of liver lobules, the liver cells were accompanied by mild edema and steatosis, apoptotic bodies were occasionally seen around the central vein, the inflammatory infiltration was significantly reduced, and the pathological manifestations of liver tissues tended to be normal (Figure 5E). The pathological changes in the HSYA-M group were between the above two groups (Figure 5D). A quantitative analysis of necrotic areas in liver tissue revealed that there was no evidence of liver cell degeneration or necrosis around the central vein of the liver in the control group. Compared with the control group, the necrotic area of liver in the model group was significantly increased (*p* < 0.01). In addition, compared with the model group, except the HSYA-L group, the necrotic area of liver in the HSYA-M and HSYA-H groups were drastically reduced (*p* < 0.05) (Figure 5F).

### 3.6 RNA-sequencing data analysis

#### 3.6.1 RNA-sequencing analysis

To elucidate the mechanism underlying how HSYA performed a protective role in ALI, three liver samples of rats were chosen at random from the control, model and HSYA-M groups (the following is represented by the HSYA group) to construct mRNA libraries. After the quality control of RNA-Seq was qualified, the sequencing data in the three groups were analyzed. As shown in Table 2, these data indicate that the sequencing data are reliable.

**TABLE 2:** RNA-seq data analysis.

Group	Clean_reads	Total_map (%)	Unique_map (%)	Q30 (%)	GC_pct (%)	Exon (%)	Intergenic (%)
Control	42515666	95.45	88.55	94.48	50.17	89.15	6.5
Model	42049237.33	95.58	89.27	94.52	50.56	88.49	7.32
HSYA	42794086	95.7	89.55	94.33	50.25	89.18	6.89

#### 3.6.2 Quantitative analysis of differentially expressed genes

**FIGURE 6:**
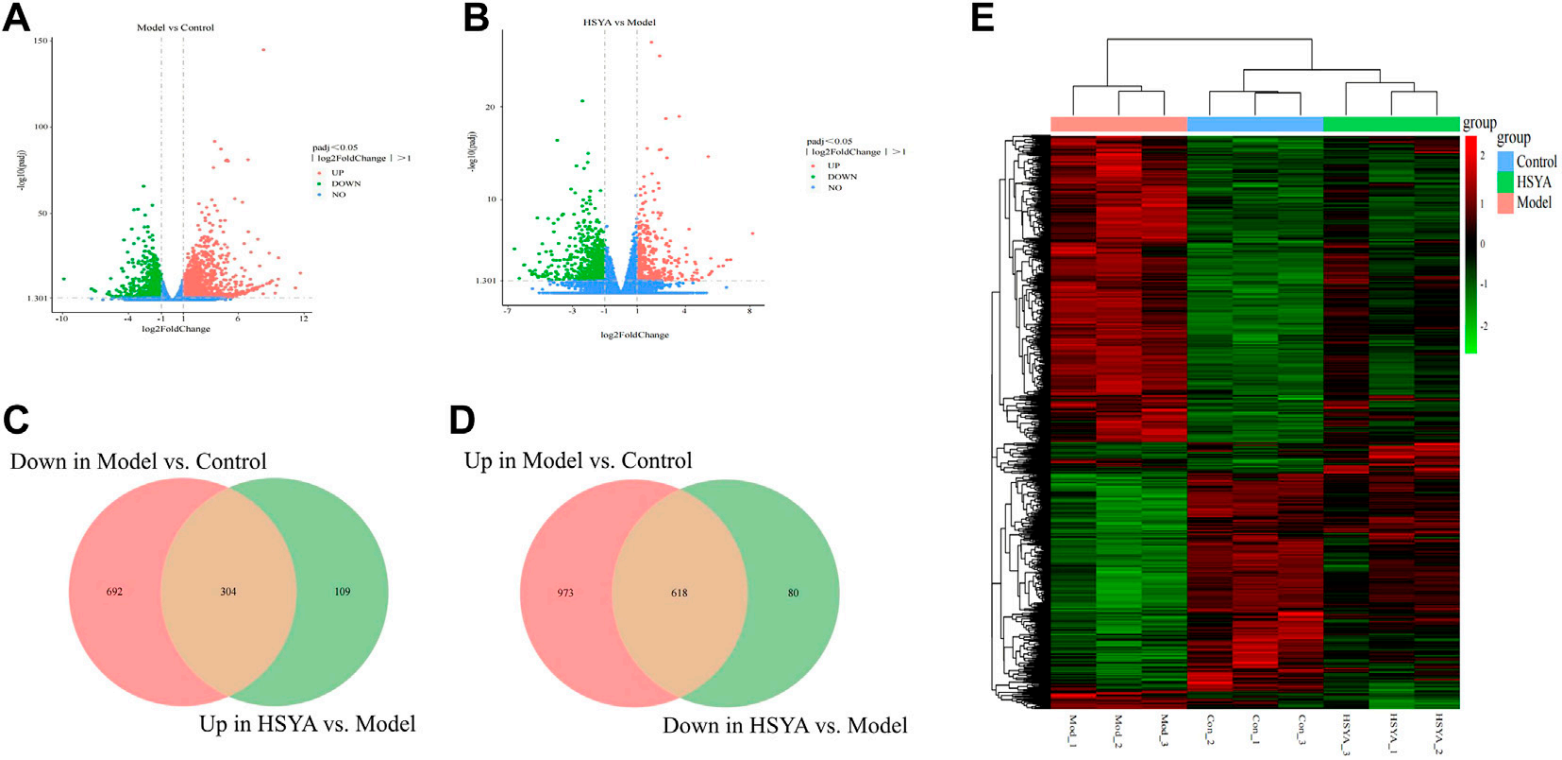
Quantitative analysis of DEGs. **(A,B)** Volcanic map of DEGs among groups; **(C,D)** Venn diagram of DEGs among groups; **(E)** Heat map among liver tissues.

We detected the patterns of gene regulation in hepatic tissue from control, model, and HSYA (HSYA-M) groups by RNA-Seq. 1,591 DEGs were upregulated and 996 were downregulated between control and model groups, whereas 413 were upregulated and 698 were downregulated between HSYA and model groups (Figures 6A,B). To elucidate the improvement mechanism of HSYA on ALI, further DEGs screening has been carried out. According to the findings, 618 genes were elevated by CCl_4_ and downregulated by HSYA. Meanwhile, 304 genes were downregulated by CCl_4_ and elevated by HSYA (Figures 6C,D). We suspected these genes are potential genes for HSYA to prevent CCl_4_-induced ALI. Cluster analysis revealed substantial differences between the three groups (Figure 6E).

#### 3.6.3 Differentially expressed genes analyzed by GO and KEGG

**FIGURE 7:**
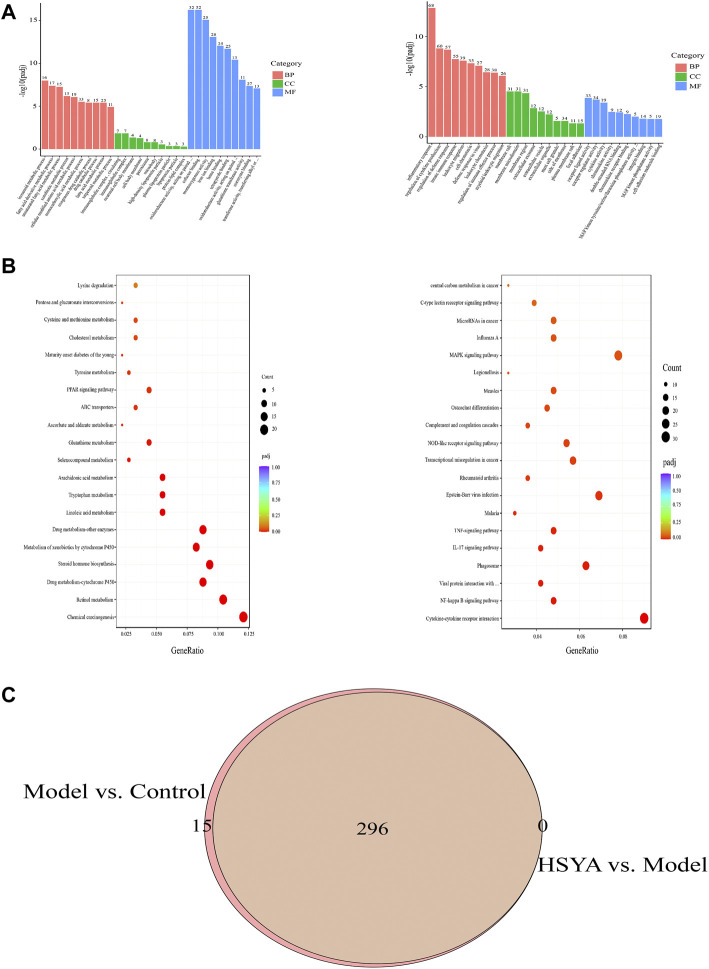
DEGs were analyzed by GO and KEGG. **(A)** Analysis of 304 upregulated DEGs and 618 downregulated DEGs by GO; **(B)** Analysis of 304 upregulated DEGs and 618 downregulated DEGs by KEGG; **(C)** 296 altered pathways by CCl4 treatment were attenuated by HSYA pretreatment.

After obtaining the DEGs of HSYA in the pretreatment of ALI, GO analysis was conducted on up- and down-regulated DEGs. The 30 most enhanced GO phrases were selected for analysis (*p* < 0.05). BP (Biological Process) analysis of the upregulated DEGs has mainly exhibited enrichment for icosanoid metabolic process, exogenous drug catabolic process, fatty acid derivative metabolic process and so on; the downregulated DEGs has mainly exhibited enrichment for inflammatory response, regulation of cytokine production, innate immune response, leukocyte migration and so on. The CC (Cellular Component) analysis revealed the upregulated DEGs were mostly found in the immunoglobulin complex, neuronal cell body membrane and peroxisome; the downregulated DEGs were mostly found in the membrane raft, membrane microdomain, membrane region, etc. In terms of MF (Molecular Function), the upregulated DEGs were mostly concentrated on cofactor binding, monooxygenase activity, iron ion binding, etc; the downregulated DEGs mainly focus on receptor-ligand activity, double-stranded RNA binding, chemokine receptor binding, etc. (Figure 7A). Additionally, we did a KEGG analysis further to investigate the pathogenic mechanism of HSYA pretreatment ALI and to identify the pathways associated with the functions between Model vs. Control and HSYA vs. Model. The top 20 signaling pathways with the greatest enrichment were selected (Figure 7B). There were 296 shared pathways among the enriched pathways between Model vs. Control and HSYA vs. Model (Figure 7C). Among the intersecting paths, Retinol metabolism, PPAR, NF-kappa B, NOD-like receptor signaling pathways were observed; it is hypothesized that HSYA pretreatments may influence ALI with the above pathways.

#### 3.6.4 qRT-PCR validation of differentially expressed genes

**FIGURE 8:**
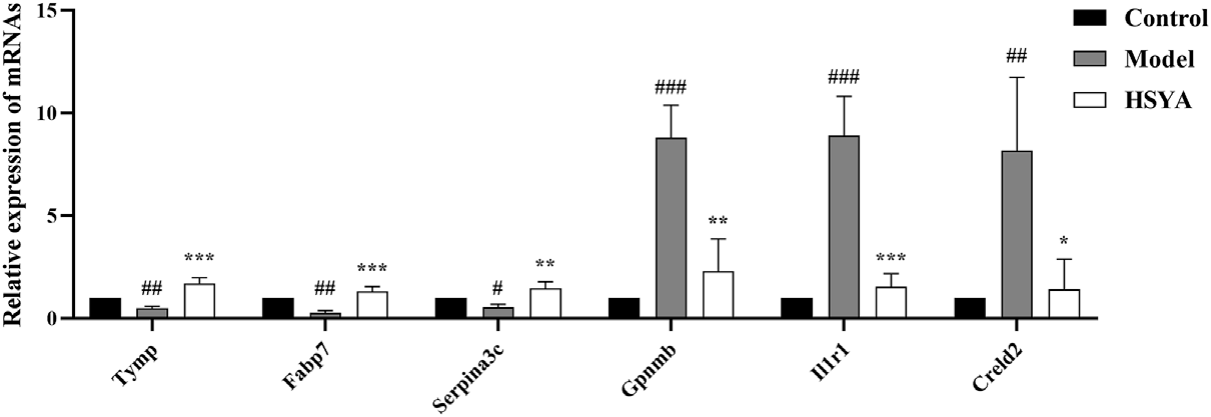
Three upregulated DEGs and three downregulated DEGs mRNA expression levels ( $\bar{x}$ ± *s*, *n* = 3). ^#^ *p* < 0.001, ^##^ *p* < 0.001, ^###^ *p* < 0.001 vs. the control group; *p < 0.05, **p < 0.01, ***p < 0.001 vs. the model group.

The genes involved in the regulation of ALI by HSYA were selected for qRT-PCR to estimate the mRNA expression levels. Screening genes according to the following principles: The *p* value should be much less than 0.05; genes ought to be highly expressed, that is, the FPKM value of each sample should be at least more than 20; 1.0 ≤ |log_2_FC| ≤ 3.0; the read count should be relatively high. Based on the above filters, a total of 91 upregulated and 28 downregulated DEGs were selected. The specifics of the top 10 DEGs that were upregulated or downregulated by HSYA administration are reported in Table 3. The first three upregulated DEGs and the first three downregulated DEGs were selected for qRT-PCR verification, the results are shown in Figure 8, compared with the control group, Tymp, Fabp7, and Serpina3c mRNA levels were diminished significantly in the model group (*p* < 0.01, *p* < 0.05), meanwhile, Gpnmb, Il1r1, and Creld2 mRNA levels elevated drastically (*p* < 0.001, *p* < 0.01, and *p* < 0.05). Tymp, Fabp7, and Serpina3c mRNA levels were considerably higher in the HSYA group than in the model group (*p* < 0.001, *p* < 0.01), Gpnmb, Il1r1, and Creld2 mRNA levels were declined substantially (*p* < 0.01, *p* < 0.05). qRT-PCR corroborated that the same pattern in the expression levels of DEGs that matched the RNA-seq data.

**TABLE 3:** The top 10 up- and down-regulated DEGs in HSYA.

Gene ID	Gene name	Log_2_FC	*p* adj
ENSRNOG00000032394	Tymp	1.896308884	1.23E-27
ENSRNOG00000000814	Fabp7	2.404946406	3.61E-26
ENSRNOG00000010410	Serpina3c	2.785841518	1.92E-19
ENSRNOG00000029993	Kynu	1.927743293	1.54E-13
ENSRNOG00000048874	Gckr	1.429194087	3.23E-13
ENSRNOG00000023116	Agmo	1.421670722	3.92E-12
ENSRNOG00000002878	Afm	1.210173494	6.98E-12
ENSRNOG00000001736	Bdh1	1.219810299	3.39E-11
ENSRNOG00000015438	Ces1f	1.527371	1.59E-10
ENSRNOG00000033010	Akr1c12	1.411245353	2.09E-10
ENSRNOG00000008816	Gpnmb	−2.20580116	7.25E-08
ENSRNOG00000014504	Il1r1	−1.367891194	4.61E-07
ENSRNOG00000004659	Creld2	−1.849881578	1.22E-06
ENSRNOG00000015078	Ifitm3	−1.146379749	3.04E-06
ENSRNOG00000026605	Ifi27l2b	−1.271733055	3.34E-06
ENSRNOG00000014117	Hmox1	−1.188417247	3.73E-06
ENSRNOG00000042499	Tmsb10	−1.300520436	5.21E-06
ENSRNOG00000049075	Fabp5	−1.077557324	6.01E-06
ENSRNOG00000007650	Cd63	−1.060319089	6.66E-06
ENSRNOG00000007089	Lgmn	−1.108294769	8.02E-06

### 3.7 Effects of hydroxysafflor yellow A on the reactive oxygen species and inflammatory genes

**FIGURE 9:**
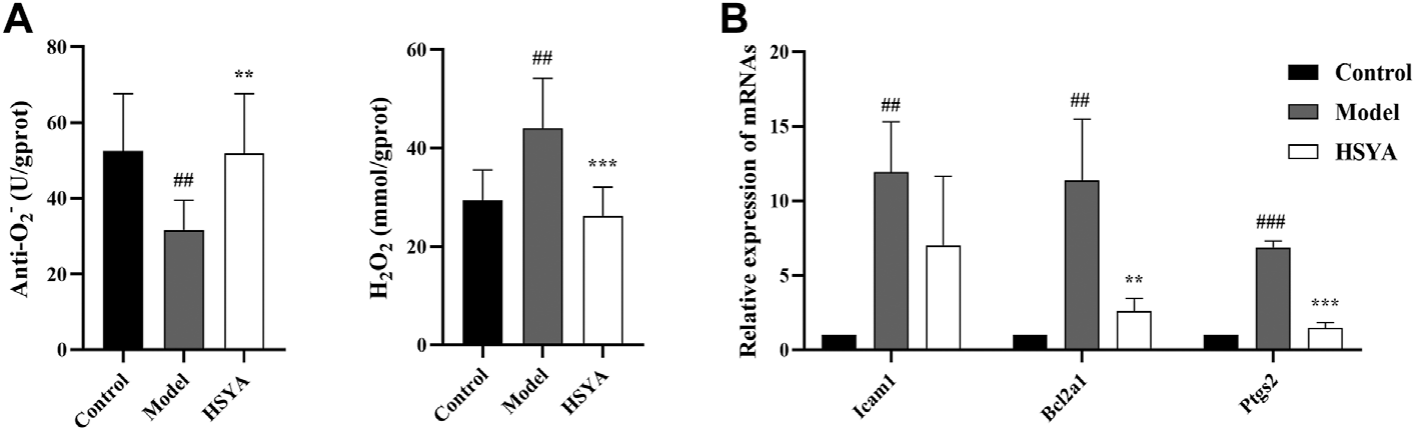
Effects of HSYA on the ROS and inflammatory genes. **(A)** The contents of Anti-O_2_^−^ and H_2_O_2_; **(B)** The expression levels of inflammatory genes ( $\bar{x}$ ± *s*, *n* = 3). ^##^ *p* < 0.001, ^###^ *p* < 0.001 vs. the control group; **p < 0.01, ***p < 0.001 vs. the model group.

As shown in Figure 9, two components of reactive oxygen species (ROS) were determined. The findings revealed that relative to the control group, the anti-O_2_^−^ content was substantially decreased (*p* < 0.01), and the H_2_O_2_ content was considerably elevated in the model group (*p* < 0.01). Relative to the model group, the anti-O_2_^−^ content was substantially increased (*p* < 0.01), and the content of H_2_O_2_ was substantially decreased in the HSYA group (*p* < 0.001) (Figure 9A). Moreover, we selected three inflammatory genes in the NF-kB signaling pathway to confirm the exact role of HSYA in CCl_4_-induced ALI. The results showed that the gene expression levels of Icam1, Bcl2a1, and Ptgs2 in the model group were significantly higher than those of the control group (*p* < 0.001, *p* < 0.01). In the HSYA group, Bcl2a1 and Ptgs2 expression levels were significantly lower than that of the model group (*p* < 0.001, *p* < 0.01) (Figure 9B). Combined with the results in Section 3.4, it was further suggested that HSYA can protect the liver from damage by inhibiting inflammatory response and oxidative stress.

## 4 Discussion

HSYA is a water-soluble monomer component extracted from safflowers that promotes blood flow, removes blood clots, relaxes and activates tendons (Bai et al., 2020). CCl_4_ model is one of the classical models for inducing ALI, which is often used to investigate hepatotoxicity and hepatic fibrosis, as well as the hepatoprotective functions of pharmaceuticals and natural substances (Zhao et al., 2020). The mechanism involves oxidative stress, lipid peroxidation of the liver membrane, inflammatory reaction, apoptosis, and necrosis of liver cells (Li et al., 2021a). Rats were used in this research to construct a CCl_4_-induced ALI model, and the liver protective activity of HSYA was investigated. Using RNA-Seq to analyze the possible effects of HSYA on ALI, provides a reliable basis for further development of HSYA as a secure and reliable medication for liver damage avoidance and therapy.

In the metabolic process of the body, CCl_4_ can generate CCL_3_ and CCl_3_O_2_ free radicals through the cytochrome P450 enzymes, which may cause a structural collapse of liver cell membranes and enhance their permeability, and large amounts of intracellular enzymes, including ALT, AST, and ALP are released into the bloodstream, resulting in liver damage (Guo et al., 2016; Susilo et al., 2019). Consequently, the measurement of serum ALT, AST, and ALP activity is an essential criterion for assessing ALI (Xie et al., 2015).

Moreover, these ROS are capable of attacking fatty acids and causing peroxidation (Li et al., 2019). As the metabolic end-product of lipid peroxides, MDA can aggravate cell membrane damage by destroying the function, structure, and metabolism of biofilms (Lixin et al., 2019). SOD is the principal antioxidative enzyme, which can transform free radicals into water and molecular oxygen, thus reducing cell damage (Wu et al., 2017). Therefore, MDA and SOD are often considered sensitive oxidative stress indicators. Meanwhile, these free radicals can induce inflammatory, with the release of inflammatory factors, which accelerate the development of liver injuries (Li et al., 2020). In this study, it was discovered that HSYA considerably decreased the blood AST, ALT, and ALP levels of CCl_4_-induced ALI in rats, alleviated the pathological alterations of liver tissue, decreased the MDA content, inflammatory factors (TNF-α, IL-1, and IL-6) levels, as well as increased the SOD activity. These findings imply that HSYA may effectively prevent rats with ALI caused by CCl_4_.

The invention of RNA-seq technology facilitates the investigation of the molecular basis of illness. Thus, RNA-Seq was undertaken to study the DEGs and associated pathways after HSYA pretreatment that might help in the prevention and treatment of ALI by HSYA. We observed that HSYA pretreatment reversed most genes changed by CCl_4_, such as Tymp, Fabp7, Serpina3c, Gpnmb, Il1r1, Creld2, etc. But, these genes have rarely been reported in ALI, so we performed qRT-PCR verification for them, and explored whether HSYA pretreatment exerts its functional role on ALI through these genes.

Tymp (thymidine phosphorylase) is a nucleoside metabolism enzyme induced by TNF-α, which is essential for angiogenesis, cell apoptosis and multiplication (Schwartz et al., 2010; Elamin et al., 2016). Earlier studies have shown that Tymp is overexpressed and associated with tumor growth in a variety of cancers (Marangoni et al., 2018; Ramadan et al., 2020; Gao et al., 2021; Poortahmasebi et al., 2022). But our research, compared to the control group, revealed that Tymp expression was considerably down-regulated in the model group, which could be reversed after HSYA intervention. The reason why the model group’s Tymp expressions were lower, despite the fact that the TNF-α expressions were greater in the model group, remains unanswered. It’s the first research to examine the Tymp expression in rats with ALI caused by CCl_4_. Although the explanation of this surprising discovery is unclear, it heavily implies that the livers of rats with ALI may contain unique Tymp downregulation that are more efficient than TNF-α. Zhao et al. (2018) confirmed that Tymp could inhibit the purine, pyrimidine, and bile acid metabolism levels. We suspected that the Tymp expression levels in rats with ALI were inhibited by an increase in thymidine demand, but further fundamental research is required to investigate this possibility.

Fabp7 is a fatty acid-binding protein of the brain type. Recently, Zhang et al. (2013) reported that Fabp7 participates in the absorption and transport of fatty acids, as well as the control of other biological functions, like signal conduction, proliferation, and differentiation. Last but not least, it also performs a key function in oxidative stress resistance; lower Fabp could induce hepatocyte injury by encouraging the production of an abundance of ROS and initiating lipid peroxidation. Prior studies have shown that the Fabp expression decreased significantly after dexamethasone feeding, and the change in Fabp level occurred before the increase of hepatic and serum cholesterol levels, demonstrating that Fabp is an indicator of early hepatic damage (Rajaraman et al., 2007; Tu et al., 2021). Several observations indicate that some hepatoprotective medications, like clofibrate, diacerein, and icariin, can enhance the antioxidative capability of damaged liver cells by inducing Fabp expression, further reducing cell apoptosis and necrosis, and improve liver function (Rajaraman et al., 2007; Liu et al., 2014; Ibrahim et al., 2021). Consistent with the above reports, the present study showed that Fabp7 was down-regulated in the model group, and this process could be reversed after HSYA intervention, suggesting that HSYA has a substantial beneficial influence against CCl_4_-induced ALI. Its mechanisms may include decreasing oxidative stress and improve Fabp7 expression.

In the rat, serpina3 was annotated as either serpina3k or serpina3c (Sánchez-Navarro et al., 2022). It is crucial for oxidative stress and inflammatory response, inflammatory response, tumor angiogenesis, apoptosis, proliferation, and migration (Yao et al., 2013; Jing et al., 2019; Ji et al., 2020; Qian et al., 2021). Some studies have shown that serpina3c participates in a series of pathophysiological processes: metabolic diseases, vascular diseases, optic nerve injury, etc. (Zheng et al., 2017; Choi et al., 2020; Qian et al., 2021). To our knowledge, until now, there has been no functional study on ALI-associated serpina3c. In the current experiment, serpina3c expression was dramatically decreased in the model group, whereas this decline was reversible with HSYA intervention. This finding suggested that HSYA may inhibit peroxidation and inflammation from protecting the liver from damage. Nevertheless, the precise mechanism may be explored deeper.

Glycoprotein nonmetastatic melanoma protein b (Gpnmb) contributes to osteoblast differentiation, inflammatory regulation, and tissue remodeling (Li et al., 2010; Abdelmagid et al., 2015; Nickl et al., 2021). Recently, some liver diseases have been associated with the expression of Gpnmb, such as ALI, cirrhosis, and alcohol-associated hepatitis (Onaga et al., 2003; Haralanova-Ilieva et al., 2005; Harris et al., 2022). Michalinos et al. (2020) found that Gpnmb was upregulated in the liver and kidneys following hepatic damage, but after treatment with hepatoprotective drugs, its expression was significantly decreased. Similar to the preceding outcomes, in this work, we observed that HSYA could drastically reduce the hepatic expression levels of Gpnmb in rats with CCl_4_-induced ALI, suggesting that this gene may be a potential target for intervention in ALI.

The interleukin-1 receptor (IL-1R) mediates several physiologic activities of interleukin-1 (IL-1) to trigger a pro-inflammatory immunological system (Anzaghe et al., 2019; Li et al., 2021b). IL1R1 is overexpressed in some liver diseases, such as ALI, liver fibrosis, and liver cancer (Gehrke et al., 2018; Dang et al., 2020; Li et al., 2021c). The present study revealed that IL1R1 expression was considerably higher in the model group than in the control group, and obviously decreased after HSYA intervention, suggesting that this gene may be utilized as a diagnostic marker for ALI.

Creld2 is a ∼50 kDa secretory glycoprotein (Oh-hashi et al., 2009). Some studies confirmed Creld2 is the crucial gene and a viable therapeutic target for hepatic steatosis and hepatocellular carcinoma (Liu et al., 2019; Kern et al., 2021). In the current research, compatible with the study of RNA-Seq, qRT-PCR suggested that HSYA can reduce Creld2 levels in rats with CCl_4_-induced ALI. Although no studies have reported the correlation between Creld2 and ALI, Creld2 was discovered to be a new ER stress-inducible gene (Oh-hashi et al., 2009). ERS is closely related to ALI (Cai et al., 2022), so we suspected that this gene is closely related to ALI, and HSYA may inhibit ERS by regulating the expression of Creld2 to protect the liver from damage.

The above transcriptomics results analyzed the DEGs in the liver for CCl_4_-induced under HSYA pretreatment. RNA-Seq and qRT-PCR studies yielded identical outcomes, supporting the validity of RNA-Seq data. We speculate that these genes may be the key genes for the diagnosis and prevention of ALI. Meanwhile, our investigation revealed that HSYA might exhibit benefits for preventing ALI *via* numerous pathways, including those linked to inflammation and lipid metabolism. Pathways involved in inflammation include the TNF, NF-kappa B, NOD-like receptor signaling pathways, and pathways involved in lipid metabolism include retinol metabolism, and PPAR signaling pathway. It has been reported that retinol metabolism and PPAR signaling pathway are closely related to acute liver injury (Chen et al., 2019; Yan et al., 2022).

According to the transcriptome results, we further explored the exact possible mechanisms of HSYA on CCl_4_-induced ALI. ROS are a group of highly reactive oxygen-containing substances, mainly including anti-O_2_^−^, hydroxyl radical (·OH ), and hydrogen peroxide (H_2_O_2_) (Moloney and Cotter, 2018). Excessive ROS can induce oxidative stress in the liver and inhibit antioxidant stress defense pathways (Zhou et al., 2019). Some studies have confirmed that CCl_4_ leads to necrosis and apoptosis of liver cells by increasing content of ROS, thus causing ALI (Xu et al., 2022). Therefore, reducing ROS contents is important for the prevention of CCl_4_-induced ALI. In this study, we found that in the model group, the content of H_2_O_2_ was significantly increased and the level of anti-O_2_^−^ was markedly decreased. However, after HSYA intervention, the phenomenon was able to be reversed. Meanwhile, the inflammatory response was researched. Inflammation plays an essential role in the process of ALI (Huang et al., 2017). Moreover, we selected three inflammatory genes in the NF-kB signaling pathway to confirm. The results showed that the gene expression levels of Icam1, Bcl2a1, and Ptgs2 in the model group were significantly higher than those of the control group. In the HSYA group, Icam1, Bcl2a1, and Ptgs2 expression levels were significantly lower than that of the model group. Combined with the above detected indicators, MDA, SOD and inflammatory factors (TNF-α, IL-1, and IL-6), it was further suggested that HSYA can protect the liver from damage by inhibiting inflammatory response and oxidative stress.

## 5 Conclusion

In a word, HSYA may prevent CCl_4_-induced ALI through a number of mechanisms, as predicted from RNA-Seq analysis and experimentally confirmed. This research demonstrated that HSYA ameliorated hepatic pathological damage and function; the possible underlying molecular mechanism is that HSYA protects the liver from damage by decreasing oxidative stress, inflammatory response, regulating the Tymp, Fabp7, Serpina3c, Gpnmb, Il1r1, Creld2, and other genes expression levels as well as the retinol metabolism, PPAR, NF-kappa B, and NOD-like receptor signaling pathways. This work may help us comprehend the course of ALI and give a new proof that HSYA prevents ALI. However, more testing is required to confirm discovered genes and pathways.

## Data Availability

The original contributions presented in the study are included in the article/Supplementary Material, further inquiries can be directed to the corresponding author.
